# A physiology-driven approach to postoperative sedation after urgent liver transplantation for acute liver failure

**DOI:** 10.1016/j.bjane.2026.844742

**Published:** 2026-03-07

**Authors:** Deepak K. Tempe, Luiz Guilherme V. da Costa

**Affiliations:** a Institute of Liver and Biliary Sciences (ILBS), Department of Anesthesiology and Critical Care, New Delhi, India; b Hospital Israelita Albert Einstein, Department of Anesthesiology and Perioperative Medicine, São Paulo, SP, Brazil

**Keywords:** Acute liver failure, Bispectral index, Cerebral autoregulation, Conscious sedation, Liver transplantation

There are no formal recommendations regarding postoperative sedation in patients with Acute Liver Failure (ALF) undergoing urgent Liver Transplantation (LT). Owing to the risk of intracranial hypertension in the perioperative period, many centers have adopted the practice of continuing propofol-based sedation for 48–72 hours after transplantation.^1^ In clinical practice, however, postoperative sedation strategies are heterogeneous and may pursue different objectives, ranging from deep sedation aimed at metabolic suppression and neuroprotection (e.g., RASS -4/-5) to lighter levels intended to facilitate airway protection, mechanical ventilation, and overall intensive care unit management. This editorial seeks to dwell into the physiological rationale that should guide the postoperative sedation in this complex clinical scenario.

A growing body of evidence indicates that many patients with ALF already exhibit profound suppression of cerebral electrical activity at the time of transplantation, with Bispectral Index (BIS) values frequently below 40 and, in some cases, approaching zero.^2^ The use of BIS has been shown to reliably track recovery of consciousness before and after LT in patients with fulminant hepatic failure,^3^ including reports of extreme cortical suppression during transplantation.^4^ Post-transplant BIS monitoring has further been explored as a tool to assess neurological recovery in acute-on-chronic liver failure patients.^5^

These observations raise an important physiological question: when cortical activity is already markedly suppressed as a consequence of severe metabolic encephalopathy, is further pharmacological suppression necessary, and does it confer additional neuroprotective benefit? This is particularly relevant because additional pharmacological suppression of cortical activity in patients with altered neurotransmission and heightened cerebral sensitivity may amplify pharmacodynamic effects, thereby increasing the risk of anesthetic overdose even at modest doses.^6^

Acute liver failure is characterized by rapid hepatic dysfunction, high-grade encephalopathy, and coagulopathy.^7^ Cerebral edema with increased Intracranial Pressure (ICP) remains the most feared neurological complication and a major contributor to mortality in this population.^8^ The pathogenesis of cerebral edema in ALF is multifactorial, involving ammonia-induced astrocytic swelling, systemic inflammation, oxidative stress, disruption of the blood-brain barrier, and impaired cerebral autoregulation.^8^ Therapeutic strategies therefore focus on minimizing cerebral edema while maintaining adequate neuroprotection. Unlike traumatic brain injury, invasive ICP monitoring is rarely employed in ALF because of severe coagulopathy and the associated risk of intracranial hemorrhage.^9^ Consequently, clinicians often rely on non-invasive surrogates, such as transcranial Doppler ultrasonography or optic nerve sheath diameter, in conjunction with clinical and physiological parameters.^10^^,^^11^

Neuroprotective measures in ALF include aggressive control of hyperammonemia, head elevation to facilitate cerebral venous drainage, avoidance of hyponatremia, maintenance of normocapnia, and the selective use of hypothermia.^1^ Early airway protection is recommended in patients with advanced encephalopathy, typically using short-acting sedative agents. However, the optimal depth and duration of postoperative sedation after LT remain poorly defined.

Propofol has traditionally been favored in this context because of its ability to reduce the Cerebral Metabolic Rate of Oxygen (CMRO_2_), with secondary reductions in cerebral blood flow leading to potential beneficial effects on ICP.^6^^,^^12^ Consequently, elective postoperative ventilation with continuous propofol sedation has become a widespread practice in patients with ALF undergoing LT. Importantly, cerebral autoregulation is frequently impaired in ALF, and reductions in CMRO_2_ do not necessarily translate into proportional decreases in cerebral blood flow or ICP. Experimental and clinical data from neurocritical care settings further suggest that neurovascular coupling may be disrupted in critically ill brains, thereby limiting the effectiveness of metabolism-based strategies for ICP control. Moreover, variability in cerebrovascular response to sedative agents has been demonstrated in neurocritical care populations, highlighting the heterogeneity of propofol’s physiological effects.^13^

The direct cerebral vasoconstrictive effect of propofol, often cited as an independent mechanism for ICP reduction, remains controversial and appears to play a secondary role relative to metabolic suppression.^6^^,^^13^ Moreover, the magnitude and direction of propofol’s effects on cerebral physiology may vary according to baseline disease severity, mean arterial and cerebral perfusion pressure targets, autoregulatory status, and ventilation strategy, particularly PaCO_2_. Such physiological heterogeneity further challenges the assumption that continued deep sedation confers uniform benefit in terms of decreasing the ICP across all patients.^6^

In unconscious patients with ALF, clinical assessment of encephalopathy severity is inherently limited. Conventional electroencephalography remains the reference standard for evaluating cerebral electrical activity but is impractical for routine perioperative use.^14^ Processed EEG monitoring, such as the BIS, derived from frontal electroencephalographic signals, has therefore been adopted as a surrogate of cortical activity. It should be emphasized, however, that BIS was primarily developed to assess the depth of anesthesia and may be influenced by encephalopathy-related EEG patterns, electromyographic activity, and artifacts. Accordingly, BIS and other processed EEG indices should be interpreted as adjunctive tools rather than stand-alone indicators of cerebral protection or neurological prognosis. Importantly, low BIS values do not directly reflect intracranial pressure status and do not exclude the presence of intracranial hypertension.

Notably, intracranial hypertension may still occur despite profound cortical electrical suppression, reinforcing the need for a multimodal approach to clinical interpretation. Hemodynamic variables, ventilation parameters, temperature, metabolic control, and available neuromonitoring surrogates should all be integrated when guiding postoperative management in this vulnerable population.

From a practical perspective, postoperative sedation after urgent LT for ALF may be approached using a physiology-guided framework aligned with established clinical recommendations. The primary goal of sedation should first be clearly defined ‒ whether airway protection and ventilatory synchrony, control of agitation, temperature management, or treatment of suspected seizures. Alongside sedation decisions, priority should be given to established multimodal neuroprotective strategies, as outlined above. When available, modern frontal EEG monitoring may assist in titrating sedative exposure and avoiding unnecessary escalation when cortical activity is already profoundly suppressed.^15^

This physiology-guided perspective should not be interpreted as an argument against postoperative sedation per se. Sedation may remain clearly indicated in several clinical scenarios after LT for ALF, including agitation compromising airway safety or causing significant ventilator dyssynchrony; suspected or evolving intracranial hypertension based on clinical course or non-invasive surrogates; suspected seizures or status epilepticus, particularly when EEG monitoring is available; and during targeted temperature management protocols.

In the absence of definitive guidelines, this editorial reasons that postoperative sedation after urgent LT for ALF should be individualized and guided by physiological principles rather than applied routinely. Based on the foregoing discussion, the authors advocate routine monitoring of BIS in all patients undergoing LT for ALF. The BIS value should be incorporated into decision-making regarding postoperative sedation, with the clear understanding that sedation should not be administered in patients who already have very low BIS values solely with the objective of lowering CMRO_2_ in an attempt to lower the ICP. In this context, processed EEG monitoring may serve as a valuable adjunct within a multimodal management framework, assisting clinicians in balancing the potential benefits and risks of continued sedative exposure.^15^ Future prospective studies integrating processed EEG, non-invasive ICP surrogates, and neurological outcomes are needed to define optimal sedation strategies in this complex and high-risk population.

## Data availability statement

No new data were created or analyzed in this study. Data sharing is not applicable to this article.

## Authors' contributions

Both authors contributed to the conception and critical discussion of the editorial. Deepak K. Tempe drafted the manuscript. Luiz Guilherme V. da Costa critically revised the manuscript for important intellectual content. Both authors approved the final version of the manuscript.

## Funding

None.

## Conflicts of interest

The authors declare no conflicts of interest.
